# Influence of genome and bio-ecology on the prevalence of genome exchange in unisexuals of the *Ambystoma* complex

**DOI:** 10.1186/s12862-018-1200-7

**Published:** 2018-05-31

**Authors:** France Beauregard, Bernard Angers

**Affiliations:** Departement of biological sciences, Université de Montréal, C.P. 6128, Succ. Centre-ville, Montréal, QC H3C 317 Canada

**Keywords:** *Ambystoma*, Genome exchange, Clonal reproduction, Kleptogenesis, Unisexual, Blue-spotted salamander-dependent populations

## Abstract

**Background:**

Unisexuals of the blue-spotted salamander complex are thought to reproduce by kleptogenesis. Genome exchanges associated with this sperm-dependent mode of reproduction are expected to result in a higher genetic variation and multiple ploidy levels compared to clonality. However, the existence of some populations exclusively formed of genetically identical individuals suggests that factors could prevent genome exchanges. This study aimed at assessing the prevalence of genome exchange among unisexuals of the *Ambystoma laterale*-jeffersonianum complex from 10 sites in the northern part of their distribution.

**Results:**

A total of 235 individuals, including 207 unisexuals, were genotyped using microsatellite loci and AFLP. Unisexual individuals could be sorted in five genetically distinct groups, likely derived from the same paternal *A. jeffersonianum* haplome. One of these groups exclusively reproduced clonally, even when found in sympatry with lineages presenting signature of genome exchange. Genome exchange was site-dependent for another group. Genome exchange was detected at all sites for the three remaining groups.

**Conclusion:**

Prevalence of genome exchange appears to be associated with ecological conditions such as availability of effective sperm donors. Intrinsic genomic factors may also affect this process, since different lineages in sympatry present highly variable rate of genome exchange. The coexistence of clonal and genetically diversified lineages opens the door to further research on alternatives to genetic variation.

**Electronic supplementary material:**

The online version of this article (10.1186/s12862-018-1200-7) contains supplementary material, which is available to authorized users.

## Background

Unisexuality in vertebrates is often considered an ephemeral anomaly from the usual course of evolution. Interspecific hybridization is the most common trigger of deviation from sexual reproduction, the standard mode of reproduction among vertebrates [1]. Most unisexual vertebrates reproduce clonally and preserve F1 heterosis acquired through interspecific hybridization. However, because of their sexually-derived ancestors, these unisexual species still produce gametes. Except in a few parthenogenetic lizards [2], most unisexual vertebrates require the sperm of a male from a closely related species to activate egg development [3].

Three modes of reproduction are known in sperm-dependent unisexuals. The most common is gynogenesis, in which the male genome is not incorporated into the unreduced genome of the eggs, making this reproduction mode totally clonal. Known unisexuals reproducing by gynogenesis include hybrids from the *Poecilia formosa* complex [4], the Poeciliopsis monacha-lucida complex [5] and the Chrosomus eos-neogaeus complex [6]. In the other two reproductive modes, females can discard a whole haploid set of chromosomes (haplome) before meiosis to produce reduced eggs that will be combined with the genome of a sexual male from a closely related species. In hybridogenesis, the male haplome is always discarded and replaced de novo, while the same female genome is conserved across generations, resulting in a semi clonal reproduction, as seen in the complex of Pelophylax esculentus (formerly *Rana esculenta*) [7]. In kleptogenesis, the female can produce unreduced or reduced eggs, and the sperm genome can be included or not. The different combinations of these events can either produce clonal offspring (as gynogenesis), ploidy elevation or reduction, or offspring with the same ploidy level as the mother but with a replaced haplome. The last scenario differs from hybridogenesis since the haplome discarded during the production of a reduced egg could be either the one inherited from the female or the one inherited from the male. The process through which unisexual reduced eggs combine with the genome of a sexual male from a given species is called genome replacement [8]. Genome replacement has been inferred from natural populations of unisexuals of the *Ambystoma laterale*-jeffersonianum complex [8, 9], but has never been demonstrated in the laboratory. Since the conserved haplomes are not the same from one generation to another, genomic turnover can limit the persistence of a clonal haplome across generations.

The genetic diversity found in unisexuals of the *Ambystoma laterale*-jeffersonianum complex is lower than that found in sexual species, but higher than that found in clonal organisms [8, 10–13]. Exclusive gynogenesis has been first proposed as the reproductive mechanism of the unisexuals of this complex [14, 15]. However, this hypothesis is not compatible with the relatively high genetic diversity detected within populations [8, 11, 16–18]. Moreover, strict gynogenesis following a single hybridization event cannot explain the presence of haplomes from distinct species in the genome of the unisexuals across their range, according to the single origin of unisexuals of the complex. All unisexual individuals share the same mitochondrial genome [19–22], but in addition to the blue-spotted salamander haplome (*A. laterale*, haplome designated “L”), unisexual individuals of this complex may include the haplome of either the Jefferson salamander (*A. jeffersonianum*, haplome “J”), the small-mouth salamander (*A. texanum*, haplome “T”), the tiger salamander (*A. tigrinum*, haplome “Ti”) or the streamside salamander (*A. barbouri*, haplome “B”).

Kleptogenesis has then been proposed to explain the relatively high genomic diversity and the presence of five different species despite the single origin of all unisexuals [8]. Since the original hybridization event, the *Ambystoma* unisexuals incorporate genetic material from the different sexual species, but the gene flow remains unidirectional. The exact mechanism of genome replacement remains unknown, and since it is difficult to assess whether haplome permutation occurs in the same reproduction event (genome replacement) or through several generations, some authors prefer to use the term genome exchange [13].

Different factors have been recognized as affecting the prevalence of genome exchange. A relatively high amount of genetic diversity was found within clutches, both in the field [8] and in the laboratory [23]. Temperature affects the prevalence of male genome inclusion leading either to genome replacement or ploidy elevation in the laboratory [23] and in the field [24]. For instance, the frequency of the male genome inclusion was higher for unisexual females kept in warm water (15 °C) between insemination and egg hatchling than for those kept in colder water (6 °C). The frequency of sperm inclusion is also expected to be dependent on the species of the sperm donor [23].

The objective of the present study was to assess the prevalence of genome exchange in the northern part of the distribution of unisexuals of the *Ambystoma laterale*-jeffersonianum complex to determine factors affecting male genome inclusion in natural conditions. To address this objective, a sampling was performed in 10 sites from Quebec (Canada), where a previous survey revealed an unusually high genetic homogeneity among individuals from the same location [25].

To address this objective, individuals were first partitioned in different evolutionary groups according to their *A. jeffersonianum* haplome. This haplome was not expected to have been exchanged recently since *Ambystoma laterale* is the only sexual species of this complex present in the region of study [26]. Genome exchange was then assessed from the *A. laterale* haplome(s) of unisexuals and sympatric sexual species *A. laterale*. Genome exchanges were expected to result in high genetic diversity for a given group within the site, in addition to a genetic similarity with sympatric *A. laterale* host individuals.

## Methods

### Sampling and DNA extraction

Sampling was conducted over two consecutive years during the reproduction period in the southern Quebec region. A total of 10 sampling sites were chosen according to the presence of Ambystoma unisexuals reported in previous surveys (personal communication of Jim Bogart, University of Guelph, and Patrick Labonté, Environment Canada; Table 1). Minnow traps were placed in ponds overnight and salamanders were collected in the morning. Because sampling success was not the same among sites, efforts were maximized in sites where the capture rate was lower. Tissue samples were collected from the tail tip of each individual and preserved in 95% ethanol. Sampling was complemented with samples previously collected by Noël et al. [25] (Table 1). However, several samples were degraded and only 14 out of 43 individuals (M06) and 11 out of 36 individuals (E01) could be analysed in the present study. DNA extraction was performed according to the phenol-chloroform purification and ethanol precipitation method of Sambrook et al. [27].

**Table 1:** Characteristics of the sampling sites and biotypes of the individuals

Site	Latitude	Longitude	Sampling year	Capture success (n/day)	Total of individuals	pure LL	LJ	LLJ	LLLJ	Total of unisexuals
Montreal region										
M01	45.392639	−73.980446	2015	6.40	32	2	7	22	1	30
M02	45.428295	−73.945999	2014–15	28.67	86	20	5	58	3	66
M03	45.441967	−73.911049	2014	0.33	2	0	0	2	0	2
M04	45.498646	−73.777199	2014	2.13	17	0	0	16	1	17
M05	45.518002	−73.73967	2014	2.38	19	0	0	18	1	19
M06	45.502444	−73.588639	2006	–	14	0	0	13	1	14
M07	45.678432	−73.511154	2014	3.14	22	6	0	16	0	16
Eastern Quebec region										
E01	45.546194	−73.169944	2006	–	11	0	11	0	0	11
E02	45.429329	− 72.62883	2014–15	1.78	16	0	16	0	0	16
E03	46.542873	−72.406937	2014–15	1.23	16	0	0	16	0	16
Total					235	28	39	161	7	207

Geographical coordinates, year of sampling, capture success, sampling size and biotypes are provided for each site. * M06 site correspond to ROY and E01 site to MSH in Noël et al.’s study [25]

To compare the site’s heterogeneity, environmental conditions were measured during the larvae development period from May to August 2014 for the sampling sites, except for M01 and M03 (Additional file 1, Table S1). Aquatic variables of the pond include conductivity, oxygen concentration, pH, oxydo-reduction potential, nature of the substratum, variation of the water level, presence of water connection, herbaceous plants and trees and percentage of tree cover above the pond. Terrestrial variables of the adjacent forest were also assessed for soil pH, drainage score, percentage of coniferous trees and the coverage level of the shrub and tree layers. A global comparison of the environmental conditions among the different sampling sites was performed using Principal Component Analysis (PCA). The relative Euclidian distance of environmental variables between sites was computed according to their scaled environmental variables.

### Genetic analyses

Genetic analyses were performed in three steps. Identification of nuclear haplomes as well as the level of ploidy was achieved using hypervariable microsatellite loci. Then, unisexual individuals were partitioned in genetic groups according to their microsatellite multiloci genotype. Individuals of the different groups were also analyzed with Amplified Fragment Length Polymorphism (AFLP) to confirm genetic differentiation among groups. Finally, the origin of mtDNA was assessed for all *A. laterale* individuals to detect eventual unisexuals whose J-haplome was switched for a L-haplome following genome exchange, and thus no longer displayed an hybrid genome [28].

### Microsatellites

Microsatellite loci were used for discrimination between the contributing sexual individuals (*Ambystoma laterale*: LL) and unisexuals (LJ, LLJ, LLLJ), as well as for determination of ploidy levels [29]. Several ploidy levels were expected in the unisexuals of the Ambystoma complex. Ploidy elevation occurs when the male genome is combined with an unreduced egg, whereas ploidy reduction occurs when no genome is included in a reduced egg. All individuals, regardless of their ploidy levels, have the potential to reproduce clonally, but the cumulative limitations of a high ploidy level seem to limit ploidy elevation [14, 30, 31].

We used eight loci amplifying the J-genome and four loci amplifying the L-genome [32, 33]. An additional set of four non-discriminatory loci were used to further assess variation, but the ranges of alleles size of J- and L-genome overlap, which hindered discrimination between species alleles (AjeD94-AjeD283) (Additional file 1, Table S2). Discriminating loci were obtained using different strategies. Three loci only amplify J-genome (AjeD13-AjeD294-AjeD378) and three others amplify alleles of both species without overlapping between alleles species (AjeD23-AjeD346-AjeD422). Four other loci were obtained by comparison of alleles amplified when using 2 distinct primer pairs, with the first pair amplifying both genomes (AjeD84b-AmaD42) and the second pair amplifying only one of the species (AjeD84 for *A. jeffersonianum*, AmaD42b for *A. laterale*).

PCR amplification was performed in a 12.5 μL reaction with approximately 30–40 ng of DNA, 1.25 μL of 10× Taq reaction buffer, 0.4 mM of dNTP mix, 0.6 mM of each primer and 0.2 U of Taq DNA polymerase. PCR consisted of an initial denaturation of 30 s at 92 °C, followed by 45 cycles of 92 °C for 30 s, 57 °C for 30 s and 68 °C for 45 s, with a final extension of 5 min at 68 °C. PCR products were run on denaturing 6% polyacrylamide (19:1 acrylamide:bis-acrylamide) gel. Silver nitrate staining was used to visualize results [34].

### Amplified fragment length polymorphism

A subsample of 71 individuals was selected to confirm genetic differentiation among groups inferred with microsatellites. AFLP analyses were performed according to a protocol modified from Xiong et al. [35]. The MspI enzyme cuts DNA regardless of methylation state at restriction site [36], so the resulting band pattern is representative of the genetic profile. The frequent cutter is MspI, but the rare cutter is KpnI. Preamplification targeted a selection of two nucleotides and involved 5’-ACGATGAGTCCTGAGCGGCC for MspI-CC extremities and 5’-GTAGACTGCGTACCGTACCGC for KpnI-GC extremities. Selective amplification required the following primers: 5’-GATGAGTCCTGAGCGGCCGC for MspI-CCGC extremities (the only used combination because of the significantly clearer results) and 5’-GACTGCGTACCGTACCGCNN for KpnI-GCNN extremities (variable combinations: GCTA, GCTT, GCAA, GCAG). At least two replicates for each combination were scored to identify loci providing reliable results. Only the bands with a clear and constant outcome between replicates were kept for analysis.

### Mitochondrial genome

The presence of unisexual mtDNA in unisexuals was confirmed using Noël et al.’s protocol [37]. Individuals presenting a LL genotype according to microsatellite DNA were also screened for the presence of unisexual mtDNA. According to Charney [28], genome replacement in unisexuals can theoretically result in individuals with an *A. laterale* nuclear genome and unisexual mtDNA.

### Statistical analyses

The genetic analyses of the unisexuals of the Ambystoma complex represent a challenge because haplome(s) from different species are present in the same individual, and individuals present different ploidy levels. In addition, dominance can occur in partial heterozygosities for polyploid individuals. For instance, triploid individuals AAB and ABB will both be genotyped AB at this locus, leaving an undetermined allele. Some functions of the package polysat for R v. 3.2 is appropriate for such datasets [38]. When the origin of the alleles is known (L or J), microsatellite data were separated into isoloci. Loci for which it was not possible to discriminate the origin of the alleles (AjeD94 and AjeD283) were not included in the polysat analysis, but they were considered for other analyses. The segregation of the homologous loci allowed the use of the function *meandist2*, which extrapolates the unknown alleles of the partial heterozygotes by weighting each possibility (ex: A or B) according to allele frequency in the population dataset.

To assess genetic distance between unisexual individuals and partition them in genetic groups according to their microsatellite multiloci genotype, we used the distance measure *Bruvo2*. This distance corresponds to the add-loss model proposed by Bruvo et al. [39], behaves according to a SMM model and allowed comparison between individuals of different ploidy levels by adding virtual alleles to the individual with the lower ploidy level. Virtual alleles are selected either among the individual with lower ploidy level (add model) or among the individual with higher ploidy level (loss model). Both possibilities were set as true. Neighbour-joining trees were built from the distance matrix with the *nj* function of the ape package in R. The trees were visualised with FigTree v 1.4.0 [40]. To assess the strength of the resulting tree, the function *bruvo.boot* of the package proppr [41] was used to perform 1000 pseudoreplicate datasets by bootstrap for the J-alleles haploid data set. Only the nodes displaying more than 50% of bootstrap value were kept on the computed tree with J-alleles. Bootstrap was not performed on the complete data set because of the various ploidy levels of the L-genome.

To assess spatial segregation of genotypes, we tested for the correlation between the abundance of genotypes among sites and geographic distance; a Mantel test was performed on Hellinger distance matrix for the abundance of genotypes with the function *mantel* in R. A Mantel correlogram was also computed between the two matrixes using the mantel.correlog function with 20 classes.

Dispersion from the centroids of the groups considering the polysat-Bruvo2 distance was used to quantify the genetic variation within groups with respect to the multiploidy constraint. The total dispersion in each genetic group was computed from the microsatellite distance matrix using the function *betadisper* of the package vegan in R [42], which returns the value of the mean distance from the centroid. To compare total dispersion between genetic groups, an ANOVA test with post-hoc Tukey HSD were then performed using the function *aov* and *HSD.test*. Dispersion intra-sites and inter-sites were also obtained and compared by the same method, with modification of the groups’ attributions.

For a comparison between unisexuals and sexual host individuals, allelic frequencies of pure individuals LL were obtained and compared to the LL part of the genome of LLJ triploid unisexuals. Allelic frequencies were computed for the pure individuals LL with Genepop on the web v. 4.2 [43]. The frequency of alleles was calculated in the same manner for the sympatric unisexuals by removing the J genome from triploids to allow comparison on a diploid basis, since they all have two sets of the L-genome. The number of alleles for each genetic group per site where *A. laterale* individuals were found was weighted to account for the different number of individuals with the function *allelic.richness* of the package hierfstat in R [44].

We first assessed genome exchange by comparing the mean number of alternative alleles between the J-specific and the L-specific loci for each genetic group per site. In the absence of the *A. jeffersonianum* species, the J-haplome is restricted to a clonal mode of transmission. This can be used as a reference to assess diversity originating from mutations. In the absence of genome exchange, a similar diversity was expected between the J-specific and the L-specific loci while genome exchanges only increase diversity for the L-specific loci. The function allelic.richness of the package hierfstat in R was used to weight the number of alleles for each genetic group per site. The number of alleles was weighted according to the number of haplomes for each specific set of loci. As an example, a group of three individuals LLJ and one individual LJ would be *n* = 7 and *n* = 4 for L- and J-specific loci, respectively. This was performed for the set of eight loci for the J-haplome (AjeD13, AjeD294, AjeD378, AmaD42-J, AjeD23-J, AjeD84-J, AjeD346-J, AjeD422-J) and the set of 4 loci for the L-haplome (AmaD42-L, AjeD23-L, AjeD84-L, AjeD422-L). The number of alternative alleles was calculated by removing the minimal number of alleles of the group from the weighted sum to control for the ploidy level. For each group’s weighted ploidy level, the group’s sum of all haplomes of each specific set of loci (L or J) was divided by the total number of individuals of the group. As an example for the L-haplome, a group of three individuals LLJ and one individual LJ would have a basic weighted ploidy level of (2 + 2 + 2 + 1)/4 = 1.75 L-alleles and (1 + 1 + 1 + 1)/4 = 1 J-alleles under strictly clonal reproduction without mutation. The mean number of alternative alleles was compared between the J-specific and the L-specific loci for each genetic group per site, since the J-haplome can be used as a reference for a scenario of clonal reproduction in the study area.

The simple-match coefficient was used to calculate the distance matrix from the presence-absence AFLP matrix. We first assessed the consistency between AFLP and microsatellite data sets using a Mantel test performed between the distance matrix for AFLP bands and the distance matrix of the corresponding individuals for microsatellites. We also validated the different clusters obtained from microsatellites by performing neighbour-joining on the AFLP dataset. The distance matrices were used to build the neighbour-joining tree, using the executor neighbor. Bootstraps were used to assess the relative support of the different groups: 1000 replicates were generated with the executor seqboot of Phylip [45]. Distance matrices were calculated with the simple-match coefficient with a loop in R. All trees obtained with the pseudoreplicates were finally merged into one consensus tree, using the executor consens with the extended method of majority rule and compared to the real tree.

## Results

**Fig. 1:**
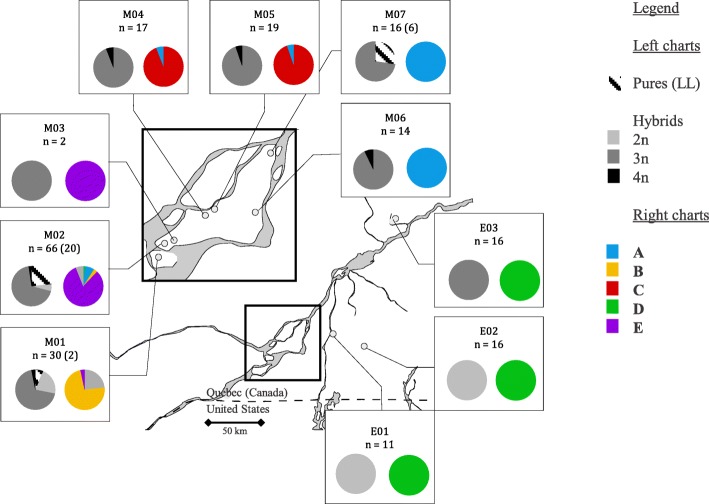
Geographic distribution and organization of the sampled unisexuals. The region in close-up represents the Montreal region. The number of unisexuals is listed beside the number of pure A. laterale individuals (the latter is in parentheses). Left pie charts: proportion of pure (LL) individuals and diploid, triploid and tetraploid unisexuals for each sampling site. Right pie charts: proportion of individuals in each main genetic group determined by the four multimodal J-loci

A total of 235 individuals were captured. Genetic analyses revealed a high proportion of unisexuals (*n* = 207, 88%) compared to *A. laterale* (LL), the unique contributing sexual species captured. The capture of *A. laterale* (LL) was restricted to three sites: (M01: n = 2; 6.25%, M02: *n* = 20; 23.3% and M07: *n* = 6; 27.3%). The unisexuals were dominated by triploids (LLJ: *n* = 161; 77.8% of all unisexual individuals), but also included diploid (LJ; 18.8%) and a few tetraploid (LLLJ; 3.38%) unisexuals (Table 1). Most of the sites included individuals of different ploidy levels, except two sites exclusively composed of diploid unisexual individuals (E01 and E02) (Fig. 1). Unisexual individuals presenting a hybrid genotype according to microsatellite data harbored the unisexual mtDNA, related to *A. barbouri* mtDNA. However, the presence of unisexual mtDNA was detected in none of the 28 individuals presenting a LL nuclear genotype.

### Inference of the genetic groups

**Fig. 2:**
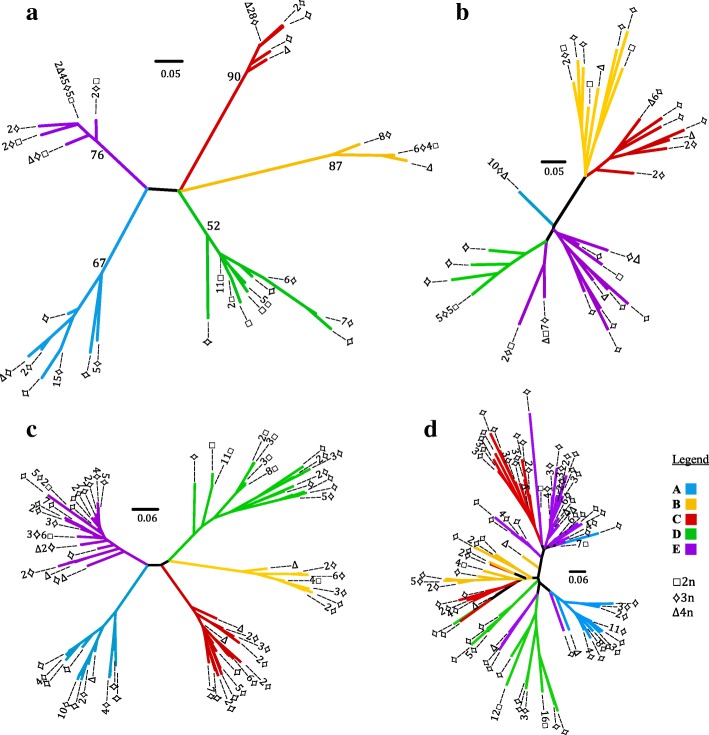
Relationships among the five main genetic groups according to different genetic data. **a** Cluster analysis of all unisexual individuals based on microsatellite data with only J alleles from eight loci. Only the nodes supported by more than 50% of the bootstrap analyses are reported (bootstrap values of the main nodes are in black). **b** Cluster analysis of 71 unisexuals based on simple match coefficient distance with AFLP data. **c** Cluster analysis of all unisexual individuals based on microsatellite data with both L and J alleles from ten loci. **d** Cluster analysis of all unisexual individuals based on microsatellite data with only L-alleles from four loci. Color represents each main genetic group. Number of individuals of each ploidy level (represented by forms) is indicated at the end of the branches

We first inferred the relationships among unisexual individuals according to the J-haplomes, since they were expected to only be transmitted clonally in the absence of *A. jeffersonianum*. Alleles of most of the J-loci were only different by one or two mutation steps, leading to a mean distance of 1.47 ± 0.62 (S.D.) mutation steps from the direct neighbours for a given allele (Additional file 1, Table S3 and Additional file 2, Sheets 1 and 8). Although all J-haplomes were highly similar, it was possible to determine five different genetic groups based on the J-alleles, since four microsatellite loci (AmaD42, AjeD378, AjeD13 and AjeD23) were characterized by large gaps among alleles without intermediate steps. For instance, the locus AmaD42J displayed a range of allele size from 133 and 193 bp, but without alleles between 137 and 177 bp. All individuals displayed the same multilocus genotype (consensus genotype), except at one or a few of these four loci. According to their multilocus genotype, individuals could be sorted in five groups (groups A to E, Additional file 1, Table S3 and Fig. 2). Individuals of group B correspond to the consensus genotype, while those of the other groups are characterized by one or a few divergent alleles from this consensus genotype (group A: AmaD42–137, group C: AjeD13–224 and AjeD23–197, group D: AjeD378–264, group E: AmaD42–193).

The AFLP analysis revealed 19 variable loci out of a total of 27 loci. A total of 17 loci were informative (at least two individuals shared the same variation) and 8 of them explained the association to a given group (Additional file 1, Table S4). Only a few nodes were supported by bootstraps higher than 50%, due to the small number of loci explaining the membership in a given group. Therefore, all nodes were kept in the computed tree for AFLP data. No variation was detected among individuals from group A, nor among diploid unisexuals of group D. Individuals presenting divergent alleles from their group were not the same from one locus to another.

The recovery of the very same clusters of individuals was apparent in the neighbour-joining trees performed with different data sets (J-haplome only, both L- and J-haplomes or AFLP) and the correlation between AFLP and LJ-microsatellites distance matrices was high (R^2^ = 0.5527; *p* < 0.001). In addition, the five genetic groups were supported by high bootstrap values for microsatellite data of the J-genome. These groups were also fully consistent with those based on multilocus genotypes and the divergent alleles of AmaD42, AjeD378, AjeD13 and AjeD23 loci. The relationships among groups slightly differed among the datasets due to the inconsistent position of group D (Fig. 2).

The individuals of these five genetic groups were also spatially structured (Fig. 1). Each site was generally characterized by individuals from a single group, but included triploid, diploid and tetraploid unisexuals. Significant positive spatial autocorrelation was detected for the first distance class (7.5 km), confirming that individuals from neighbouring sites belong to the same genetic group (R^2^ = 0.3381; *p* = 0.0406).

A high diversity of groups was observed in the Montreal Island where the presence of four of the five groups was detected. The R of Mantel between the geographic distances and the abundance of each group was not significant (*p* = 0.083); it became significant only when the sites from the Montreal region were considered (R^2^ = 0.4783, *p* = 0.004). The D group was the only group detected outside the Montreal region. Moreover, this group was also characterized by complete allopatry between diploid (sites E01 and E02) and triploid (site E03) unisexuals (Fig. 1).

The environmental data measured were not indicative of the genetic composition of the unisexual population found within a given site (Additional file 1, Figure S1). Sampling sites were comparable, except for E02 and M06. The pond of M06 site had almost dried out in July, affecting the aquatic parameters. While most sites were forest wetlands, the E02 site was more coniferous and the pond was well delimited and deeper, with a lot of herbaceous plants.

### Prevalence of genome exchange

In the absence of *A. jeffersonianum*, unisexuals were expected to use the sperm of sexual species *A. laterale* to trigger the development of eggs. Consequently, genome exchange events can only result in changes of alleles associated with L-haplomes. We therefore sought similarity between the alleles of unisexuals and those of sympatric population of sexual species *A. laterale*.

Most of the loci in the unisexuals displayed a narrow range for L-alleles, which hindered the determination of whether it was high mutation rates or genome exchange that was responsible for this genetic variation. The cluster of the five genetic groups previously determined according to J-alleles was indeed far less defined when only L-alleles were considered (Fig. 2d). Since only distinguishable L-alleles could be included in the analysis, the tree for L-alleles was built with only four loci and the analysis was more sensitive to small variations on a single locus.

Despite the narrow range of sizes for the majority of the L-alleles across the unisexuals analyzed, two loci (AjeD94-LJ and AmaD42-L; Table 2) displayed highly divergent alleles departing from the global allelic distribution (8 to 11 mutation steps from the nearest allele of the global distribution: Additional file 2, Sheet 8). Those alleles were found in the B, C and E groups and, to a less extent, in the D-3n group. Populations of the sexual species *A. laterale* also displayed these alleles at high frequency (Table 2 for the triploid results). However, none of the individuals of the A or the D-2n groups displayed these alleles (except for the tetraploid of the group A), regardless of whether it was sympatric with other unisexuals harboring those highly divergent alleles (Table 2). No diploid unisexual from any group displayed these highly divergent alleles (Additional file 2, Sheet 1). The mean frequency of individuals harboring an highly divergent allele in groups B, C, D-3n and E was 29.7% for AjeD94 and 28.2% for AmaD42 (average 28.95%), while it was 0% for both loci of group A. According to these allele frequencies, the probability of not sampling individuals with highly divergent alleles on the 36 individuals of the group A was extremely low: (1–0.2895)^36 = 4.53 E -06.

**Table 2:** Number of triploid unisexual (LLJ) and sexual individuals (LL) found and the related frequency of highly divergent alleles

		A	B	C	D	E	LL
M01	n		13			3	2
AjeD94-LJ	%		**38.5**			**0.00**	**25.0**
AmaD42-L	%		**46.2**			**0.00**	**50.0**
M02	n	5	1		1	47	20
AjeD94-LJ	%	**0.00**	**50.0**		**0.00**	**38.5**	**77.5**
AmaD42-L	%	**0.00**	**50.0**		**0.00**	**27.1**	**82.5**
M03	n					2	
AjeD94-LJ	%					50.0	
AmaD42-L	%					25.0	
M04	n	1		15			
AjeD94-LJ	%	0.00		13.3			
AmaD42-L	%	0.00		50.0			
M05	n	1		17			
AjeD94-LJ	%	0.00		11.8			
AmaD42-L	%	0.00		26.5			
M06	n	13					
AjeD94-LJ	%	0.00					
AmaD42-L	%	0.00					
M07	n	16					6
AjeD94-LJ	%	**0.00**					**100**
AmaD42-L	%	**0.00**					**20.0**
E03	n				16		
AjeD94-LJ	%				31.3		
AmaD42-L	%				3.1		
Total	n	36	14	32	17	52	28
AjeD94-LJ	%	**0.00**	**39.3**	**12.5**	**29.4**	**37.5**	**78.6**
AmaD42-L	%	**0.00**	**46.4**	**37.5**	**2.9**	**26.0**	**66.1**

For both loci AjeD94-LJ (allele 142) and AmaD42-L (alleles 241–253), the triploid unisexual and sexual individuals are organized by their J-genome affiliation (ABCDE for unisexuals and LL for sexual individuals) and by their site of origin (M01 to M07 and E03). The total number of individuals sampled (n) and the percentage of highly divergent alleles among all possible alleles (%) is given. Bold characters indicate when comparison with sexual individuals was possible

**Fig. 3:**
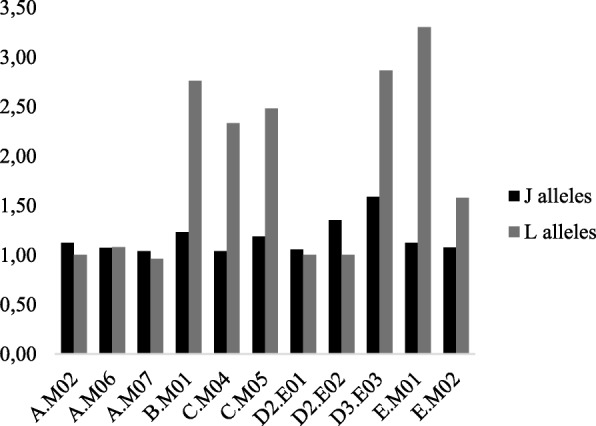
Comparison of the number of alternative alleles per locus between J and L alleles. Data are shown by subgroups, combining the identity of the genetic group (A to E) and the sampling site origin (M01 to E03) of the individuals. Data for subgroups of less than 5 individuals are not shown. Variation in ploidy levels within the population was considered

Dispersion from the centroid, used as a descriptor of the genetic variation, revealed a gradient from group E (lower total dispersion) to group D-3n (higher total dispersion) (Table 3). However, there were two distinct trends when partitioning the genetic variance within and among sites. Both the A and D-2n groups had higher genetic variation among sites than within site for the LJ-alleles, as expected from genetic variation due to mutation (Table 3). In addition, these groups were characterized by a similar number of alternative alleles per locus between J and L-haplomes (Fig. 3). In contrast, the B, C, D-3n and E groups had higher genetic variation within site than among sites (Table 3). The number of alternative alleles per locus was also 2 times higher for the L-alleles of the B, C, D-3n and E groups than for the J-alleles (Fig. 3), suggesting that mutations are not the unique source of variation for the L-haplome.

**Table 3:** Distribution of the genetic diversity among and within sites for the five main groups

Genetic groups	A	B	C	D-2n	D-3n	E
Total genetic dispersion	0.0919	0.0989	0.0940	0.1216	0.1664	0.0621
	bc	bc	bc	ab	a	c
Inter-sites dispersion	0.0741 (80.6%)	0.0049 (5.0%)	0.0050 (5.3%)	0.0978 (80.4%)	0.0219 (13.1%)	0.0048 (7.7%)
	a	b	b	a	b	b
Intra-sites dispersion	0.0178 (19.4%)	0.0939 (95.0%)	0.0891 (94.7%)	0.0238 (19.6%)	0.1445 (86.9%)	0.0573 (92.3%)
	d	b	b	d	a	c

Group D was separated between diploids and triploids because it was the only case where these biotypes were exclusively allopatric. Genetic diversity was measured by the dispersion of the individuals from the centroid based on the polysat distance accounting for the number of mutation steps. Groups designated by different lower case letters are significantly different (results of Tukey test)

## Discussion

### Recently diverged J-lineages

The very low variation detected over all J-specific markers contrasts with the high variation detected among unisexuals sampled in the geographic distribution of *A. jeffersonianum* [11, 16–18]. The diversity of the J-genome of the lineages detected in the present study is consistent with a clonal transmission of the J-haplome.

Nevertheless, five genetically distinct groups of unisexuals were detected in the studied region. Both microsatellite and AFLP loci consistently supported these groups. The low genetic variation and the equidistance between the groups suggest that the J-genome started to diverge at the same time for all groups. The geographic structure of unisexuals indicates that this genome was already different at time of colonization. However, it was not possible to assess whether this genome diverged in unisexuals or in the *A. jeffersonianum* sexual species.

In the Montreal region, distant sites exhibit different groups, while outside Montreal, the very same group can be detected in sites farther away from each other. Such distant sites harboring the same group indicates that the J-genome was already differentiated at the time of colonization, as observed in other unisexual complexes [46]. It is unlikely that random drift occurring on an assemblage of different lineages resulted in such a geographic structure. Even if migration can occur among sites, this process is not expected to occur very often given the fact that each site were largely dominated by a given genetic group, and more than half of the sites are represented by a single group.

### Variation in prevalence of genome exchange

The lack of correlation between the dendrograms of L- and J-genomes indicates that the L-genome does not seem to follow the same pattern of transmission as the J-genome. Two different patterns emerged from these five genetic groups. Groups A and D-2n displayed variation consistent with a clonal reproduction for both the L- and J-genomes. However, the higher diversity of the L-genome than the J-genome suggests that additional mechanisms to mutations would be responsible for the genetic variation of groups B, C, D-3n and E.

While a similar level of variation was detected within each group for the L-genome, there was a clear demarcation in the distribution of this variation. The genetic diversity of the A and D-2n groups was mostly found among populations as expected for isolated clonal populations. In contrast, the genetic diversity of groups B, C, D-3n and E was mainly found within populations. Moreover, the high diversity of the L-genome suggests that it gained genetic diversity faster than the J-haplome, which was expected to be clonally transmitted in the area.

Groups B, C, D-3n and E also displayed highly divergent alleles. The presence of highly divergent alleles at some loci could be explained by large mutation steps. However, these alleles are identical in state to the most frequent alleles detected in sympatric population of *A. laterale* sexual species. Their presence in some unisexuals is likely the result of genetic exchange with local pure-LL populations. Furthermore, these highly divergent alleles were segregated randomly in individuals from groups B, C and E at the different loci. This ruled out the probability that these very same divergent alleles randomly appeared by mutation, but suggests that genome exchange is one of the mechanisms responsible for intra-site variation in groups B, C, D-3n and E.

### Hypothesis for exclusively clonal reproduction

Several lines of evidence indicate that groups A and D-2n displayed a typical pattern of clonal reproduction. First, the two groups have very low variation within a given site. This variation was likely due to mutations, since the different microsatellite multilocus genotypes of a given location are separated by a single mutation step. Despite the low diversity over all individuals, groups A and D-2n displayed the major portion of their genetic diversity among sites suggesting the accumulation of mutations in isolated populations. Moreover, none of these groups displayed the highly divergent alleles found in sexual species populations, except for the tetraploid individual, for which the supplementary L-haplome seemed to come from a sexual *A. laterale* individual. Finally, the allelic diversity of the L-haplomes, when represented by the mean number of alternative alleles per locus, was also comparable to the diversity of the J-haplome, which is transmitted clonally in the studied region. This is a pattern expected from organisms reproducing exclusively clonally. The two populations with substantial amounts of unisexual individuals sampled by Noël et al. [25] were exclusively formed of unisexuals from the presumed clonal groups, A (M06 population) and D-2n (E01 population), leading to the false impression that all unisexual populations in Quebec exclusively reproduce clonally. Their results from M06 (*n* = 43 unisexuals) and E01 (*n* = 36) presented a single genotype per site for loci AjeD23, AjeD37 and AjeD422. No more than three genomotypes in low abundance and differing by a single mutation step, were found for loci AjeD94, AjeD283 and AjeD346.

Since the triploids are the most common biotype in the complex and ploidy elevation is more frequent than ploidy reduction [9, 10], all-diploid populations are unexpected. Obligatory clonal reproduction for group D-2n appears as a consequence of bioecological conditions. These all-diploid unisexual populations (E01 and E02) are exclusively composed of individuals assigned to group D. Individuals from this group can harbor a triploid genome and perform genome exchange, as observed in the E03 population which is formed exclusively of triploid individuals of the group D. The geographic segregation of the E01 and E02 populations suggests that their odd genetic composition is due to ecological causes: the only locally available sperm-donor may be *A. maculatum* [25]. No sexual *A. laterale* individuals were found at those sites, despite an extensive sampling effort in the E01 population (personal communication from Patrick Labonté, Environment Canada, [25]).

*Ambystoma maculatum* is not part of the complex, but its sperm can trigger the development of unisexuals’ eggs in the laboratory [23]. However, the inclusion of an *A. maculatum* haplome in the genome of an unisexual offspring seems unviable [23]. In the field, an extensive survey of a pond in Ontario (Canada) lead Bogart et al. [47] to conclude that LJJ unisexuals do not use sperm from *A. maculatum*. They proposed that *A. maculatum* males can discriminate between their conspecific and LJJ unisexuals, preventing them to access to *A. maculatum* sperm and reproduce [47]. In absence of a compatible sperm donor in that population, this unisexual population faces extinction, as indicated by the lower capture rate over the years [47].

The context is however different for E01 and E02 populations. Since distant E03 populations displayed the same genetic profile than E01 and E02 populations, one can expect they persist since post glacial expansion. Although this is speculative, it is possible that male *A. maculatum* do not discriminate diploid unisexuals as much as they do with LJJ unisexuals, allowing this diploid lineage to persist by gynogenesis. Reproduction by gynogenesis with *A. maculatum* is for now the most plausible explanation for the exclusively clonal and diploid populations found in E01 and E02 sites, since any attempt for genome exchange or ploidy elevation would lead to unviability.

However, ecological reasons can hardly be invoked for the clonal reproduction of individuals from the A group (from sites M06, M07 and, less abundantly, from sites M02, M04 and M05), since they coexist with sympatric sexual *A. laterale* populations. Since none of the unisexuals of group A displayed the highly divergent alleles, although the frequency of those alleles was high in the sympatric *A. laterale* populations, there was no evidence of genetic exchange with the sexual species. Indeed, all individuals from group A were highly similar, regardless of their sites of origin (M02 and M04 to M07). Individuals from group A were also detected in sympatry with those from groups B and E in the M02 population. Since individuals from groups B and E of this site seem to have acquired those highly divergent alleles by genome exchange, the lack of exchanges between the group A and the sympatric populations of *A. laterale* cannot be related to environmental conditions, as is proposed for diploid unisexuals from group D.

Furthermore, the two sites where only individuals of group A were found (M06 and M07) were ecologically very different, relative to the other sites (Additional file 1, Figure S1). The only sperm donor in the Montreal populations seems to be *A. laterale*, because *A. jeffersonianum* is absent and *A. maculatum* is either absent or very rare in this region (sampling of the present study, A.A.R.Q. [48]). It seems that group A reproduces clonally due to some intrinsic conditions that remain to be determined. Inclusion of genomes from sperm and ploidy elevation is possible and viable, since tetraploid individuals were found. A possible explanation is that ploidy reduction cannot be achieved during oogenesis in individuals of this group, preventing genome exchange.

### The genome replacement theory

Genome replacement is a mechanism suggested by several authors who have studied the similarity between unisexuals and local sexual species [8, 11, 18, 26]. Their hypotheses were fueled by the finding of a relatively high allelic diversity for a non-sexually reproducing organism, either within populations [11] or even within clutches [8], which is not consistent with an exclusively clonal mode of reproduction.

It is not possible to distinguish the resulting effect of genome replacement that occurs throughout a single reproductive event from subsequent ploidy elevation and reduction processes performed in distinct generations. Although these two hypotheses are not mutually exclusive, a certain prevalence of genome replacement seems necessary to explain the genetic variability observed in this study: the very low abundance of diploid unisexuals and tetraploid individuals in most populations makes the subsequent ploidy elevation and reduction hypothesis unlikely to explain the genetic diversity found within some of the main genetic groups.

Recombination is also a mechanism that can reorganize genetic variation among the complex. It has been observed that recombination occurs between homologous chromosomes, and more rarely between homeologs [10, 17]. It was thought that recombination would be favored by the production of reduced eggs. Both recombination and ploidy reduction are less frequent in *A. laterale* populations than in *A. jeffersonianum* populations [10]. Recombination could lead to the different combinations of highly divergent alleles found in groups B, C, and E. However, recombination alone would have to act on alleles previously present in the genome of the unisexuals. The disappearance of highly divergent alleles in all individuals of groups A and D-2n would be unlikely just by chance, since the same pattern was observed at two different loci. Moreover, the alleles absent from groups A and D-2n and present in the other groups were in high frequency in the genome of the sampled *A. laterale* individuals. Even if both hypotheses are not mutually exclusive, the hypothesis of gene flow from *A. laterale* host individuals to the unisexuals is more parsimonious to explain the different patterns observed between the groups than a hypothesis of recombination alone.

Another mechanism that can explain the nuclear diversity of the *Ambystoma laterale*-jeffersonianum complex was proposed by Bogart and Bi [9]. The occurrence of rare symmetric tetraploids (LLJJ), which would produce reduced eggs (LJ) by a regular meiotic process, without any premeiotic endoduplication, could be responsible for the haplome diversity found in this complex. In this case, the symmetric tetraploid would be created from a triploid individual that includes the haplome of the sexual species in minority in its genome (e.g., LJJ + L), therefore explaining the similarity with local sexual populations. This explanation is, however, unlikely in the present study, because all triploids are LLJ and *A. jeffersonianum* is absent from the sampled area.

A major criticism of the genome replacement theory is the absence of LL or LLL individuals with the unisexual mitochondrial haplotype [28]. Research tends to indicate that no particular haplomes were preserved in the genome, and any haplome can be replaced [17]. Considering the various combinations of haplomes already found among the complex [8, 10] and that gene flow is unidirectional between sexual and unisexual populations, it has been pointed out that we should find individuals with a nuclear genome belonging to one local sexual species coupled with the unisexual mitochondria [28]. Such combination of genomes has not yet been found, including in the present study. Either the genome replacement theory needs refinement or there is selection pressure to keep the hybrid genome [28]. Bi et al. [17] have proposed that a hybrid nuclear genome is necessary to properly operate with the unisexuals’ mitochondrial genome. They also pointed out an important restriction to genome replacement: the W chromosome being responsible for the determination of female gender. A loss of this chromosome through genome exchange cannot lead to a persistent lineage of unisexuals, unless a very specific crossing-over occurred during the production of reduced eggs [17]. Further cytogenetic analyses on the production of reduced eggs and controlled insemination followed by studies of eggs masses, would be required to determine the frequency, mechanism and requirements of genome replacement.

## Conclusion

Lineages located in the northern part of the distribution of unisexuals of the *Ambystoma* complex revealed high variability in the prevalence of genome exchange, as well as different factors preventing the occurrence of this process. Although occasional, this capacity to acquire genetic material from sexual species can provide higher genetic variation and an evolutionary advantage to kleptogens. It is therefore striking that strictly clonal unisexual lineages can coexist with genetically diversified kleptogenetic lineages, unless they have an advantage of their own.

## Additional files

Additional file 1: **Table S1.** Description of the environmental variables collected. **Table S2.** Primers and ranges of the loci used in the study. Sequence of the primers, specific range of alleles when known and reference are given. **Table S3.** Consensus score of the J-alleles for the five main genetic groups. **Table S4.** Allelic frequency for the 8 AFLP loci explaining the association to each group. **Figure S1.** Distribution of the sampling sites according to environmental conditions. (DOCX 241 kb)

Additional file 2: Microsatellite and AFLP scores of sampled individuals. **Sheet 1:** Unisexuals’ Microsatellite scores. Individuals are also associated to their main genetic group (according to its J-alleles: A, B, C, D or E – X stands for any different genotype), to a specific genotype (to acknowledge for different ploidy level or variation of L-alleles within a main group, and to associate clones) and to its biotype (ex: LLJ). **Sheet 2:** AFLP scores. The presence/absence of the different bands are labelled according to the combination used (GCNN) and the size of the band. **Sheet 3:** Polysat Matrix. Microsatellite scores in polysat format. **Sheet 4:** Polysat POP. Two matrices for population assignment in polysat: according to the sampling site or according to the main genetic group of each individual. **Sheet 5:** Matrices for weighting J. Matrices used to weight J-alleles and J-genotype. **Sheet 6:** Matrices for weighting LJ. Matrices used to weight LJ-alleles and LJ-genotype. Individual lines have been multiplied to account for ploidy variation. **Sheet 7:** LL alleles. Microsatellite scores of the LL individuals found, in table and in population format. **Sheet 8:** Statistics on m-sat scores. Allelic means, standard deviation and other parameters for each loci, including differences between alleles and the mean and its nearest neighbours, per alleles, per locus. (XLSX 181 kb)

## Abbreviations

AFLP: Amplified Fragment Length Polymorphism
DNA: Deoxyribonucleic Acid
F1: Hybrid of First generation
PCA: Principal Component Analysis
PCR: Polymerase Chain Reaction
SMM: Stepwise Mutation Model

## References

[1] Vrijenhoek RC, Dawley RM, Bogart JP (1989). Genetic and Ecological constraints on the origins and establishment of unisexual vertebrates. Evolution and ecology of unisexual vertebrates.

[2] Darevsky I. Natural parthenogenesis in a polymorphic group of caucasian rock lizards related to Lacerta saxicola eversmann. J Herpetol. 1966:115–52.

[3] Schlupp I. The evolutionary ecology of gynogenesis. Annu Rev Ecol Syst. 2005:399–417.

[4] Hubbs CL, Hubbs LC (1932). Apparent parthenogenesis in nature in a form of fish of hybrid origin. Science.

[5] Schultz RJ (1969). Hybridization, unisexuality, and polyploidy in the teleost Poeciliopsis (Poeciliidae) and other vertebrates. Amer Nat.

[6] Dawley RM, Schultz RJ, Goddard KA. Clonal reproduction and polyploidy in unisexual hybrids of *Phoxinus eos* and *Phoxinus neogaeus* (*Pisces*; *Cyprinidae*). Copeia. 1987:275–83.

[7] Berger L (1973). Systematics and hybridization in european green frogs of *Rana esculenta* complex. J Herpetol.

[8] Bogart JP, Bi K, Fu J, Noble DW, Niedzwiecki J (2007). Unisexual salamanders (genus *Ambystoma*) present a new reproductive mode for eukaryotes. Genome.

[9] Bogart J, Bi K (2013). Genetic and genomic interactions of animals with different ploidy levels. Cytogenet Genome Res.

[10] Bi K, Bogart J, Fu J (2009). An examination of genomic exchanges in *A. laterale*-dependent unisexual salamanders in the genus *Ambystoma*. Cytogenet Genome Res..

[11] Ramsden C (2008). Population genetics of *Ambystoma jeffersonianum* and sympatric unisexuals reveal signatures of both gynogenetic and sexual reproduction. Copeia.

[12] Charney ND, Ireland AT, Bettencourt BR (2014). Mapping genotype distributions in the unisexual *Ambystoma* complex. J Herpetol.

[13] Gibbs L, Denton R (2016). Cryptic sex? Estimates of genome exchange in unisexual mole salamanders (*Ambystoma* sp.). Mol Ecol.

[14] Elinson RP, Bogart JP, Licht LE, Lowcock LA (1992). Gynogenetic mechanisms in polyploid hybrid salamanders. J Exp Zool.

[15] Spolsky C, Phillips CA, Uzzell T. Gynogenetic reproduction in hybrid mole salamanders (genus *Ambystoma*). Evolution. 1992:1935–44. 10.1111/j.1558-5646.1992.tb01179.x 28567774

[16] Lowcock LA, Bogart JP (1989). Electrophoretic evidence for multiple origins of triploid forms in the *Ambystoma laterale–jeffersonianum* complex. Can J Zool.

[17] Bi K, Bogart JP, Fu J (2008). The prevalence of genome replacement in unisexual salamanders of the genus *Ambystoma* (*Amphibia*, *Caudata*) revealed by nuclear gene genealogy. BMC Evol Biol.

[18] Bogart J, Bartoszek J, Noble D, Bi K (2009). Sex in unisexual salamanders: discovery of a new sperm donor with ancient affinities. Heredity.

[19] Kraus F, Miyamoto MM (1990). Mitochondrial genotype of a unisexual salamander of hybrid origin is unrelated to either of its nuclear haplotypes. PNAS.

[20] Hedges SB, Bogart JP, Maxson LR (1992). Ancestry of unisexual salamanders. Nature.

[21] Spolsky C, Phillips C, Uzzell T (1992). Antiquity of clonal salamander lineages revealed by mitochondrial DNA. Nature.

[22] Robertson AV, Ramsden C, Niedzwiecki J, Fu J, Bogart JP (2006). An unexpected recent ancestor of unisexual *Ambystoma*. Mol Ecol.

[23] Bogart JP, Elinson RP, Licht LE (1989). Temperature and sperm incorporation in polyploid salamanders. Science.

[24] Teltser C, Greenwald KR (2015). Survivorship of ploidy-variable unisexual *Ambystoma* salamanders across developmental stages. Herpetologica.

[25] Noël S, Labonté P, Lapointe F-J (2011). Genomotype frequencies and genetic diversity in urban and protected populations of blue-spotted salamanders (*Ambystoma laterale*) and related unisexuals. J Herpetol.

[26] Bogart JP, Klemens MW (2008). Additional distributional records of *Ambystoma laterale*, *A. jeffersonianum* (*Amphibia*: *Caudata*) and their unisexual kleptogens in northeastern North America. Am Mus Novit.

[27] Sambrook J, Fritschi E, Maniatis T (1989). Molecular cloning: a laboratory manual.

[28] Charney ND (2012). Relating hybrid advantage and genome replacement in unisexual salamanders. Evolution.

[29] Ramsden C, Bériault K, Bogart JP (2006). A nonlethal method of identification of *Ambystoma laterale*, *A. jeffersonianum* and sympatric unisexuals. Mol Ecol Notes.

[30] Bogart JP, Licht LE (1986). Reproduction and the origin of polyploids in hybrid salamanders of the genus *Ambystoma*. Can J Genet Cytol.

[31] Phillips CA, Uzzell T, Spolsky CM, Serb JM, Szafoni RE, Pollowy TR. Persistent high levels of tetraploidy in salamanders of the *Ambystoma jeffersonianum* complex. J Herpetol. 1997:530–5.

[32] Julian SE, King TL, Savage WK (2003). Novel Jefferson salamander, *Ambystoma jeffersonianum*, microsatellite DNA markers detect population structure and hybrid complexes. Mol Ecol Notes.

[33] Julian S, King T, Savage W (2003). Isolation and Characterization of novel tetranucleotide microsatellite DNA markers for the spotted salamander, Ambystoma maculatum. Mol Ecol Notes.

[34] Benbouza H, Jacquemin J-M, Baudoin J-P, Mergeai G (2006). Optimization of a reliable, fast, cheap and sensitive silver staining method to detect ssr markers in polyacrylamide gels. Biotechnol Agron Soc.

[35] Xiong L, Xu C, Maroof MS, Zhang Q (1999). Patterns of cytosine methylation in an elite rice hybrid and its parental lines, detected by a methylation-sensitive amplification polymorphism technique. MGG.

[36] Jaenisch R, Bird A (2003). Epigenetic regulation of gene expression: how the genome integrates intrinsic and environmental signals. Nature Genet.

[37] Noël S, Dumoulin J, Ouellet M, Galois P, Lapointe F-J (2008). Rapid identification of salamanders from the *jefferson* complex with taxon-specific primers. Copeia.

[38] Clark LV, Jasieniuk M (2011). Polysat: an R package for polyploid microsatellite analysis. Mol Ecol Res.

[39] Bruvo R, Michiels NK, D’Souza TG, Schulenburg H (2004). A simple method for the calculation of microsatellite genotype distances irrespective of ploidy level. Mol Ecol.

[40] Rambaut A. Figtree, a graphical viewer of phylogenetic trees. Version 1.4. 2007. http://tree.bio.ed.ac.uk/. Accessed 22 Aug 2013.

[41] Kamvar ZN, Tabima JF, Grünwald NJ (2014). Poppr: an r package for genetic analysis of populations with clonal, partially clonal, and/or sexual reproduction. PeerJ.

[42] Oksanen J, Blanchet FG, Kindt R, et al*.* Vegan: community ecology package. R package version 2.3-5. 20162015.

[43] Raymond M, Rousset F. Genepop: Population genetics software for exact tests and ecumenicism. Version 1.2. In. J Hered. 1995; genepop.curtin.edu.au/. Accessed 21 Apr 2015

[44] Goudet J, Jombart T (2014). Hierfstat: Estimation and tests of hierarchical f-statistics. R package version.

[45] Felsenstein J. Phylip phylogeny inference package. Distributed by the author. Version 3.6. 2005. http://evolution.genetics.washington.edu/phylip.html. Accessed 30 Apr 2015.

[46] Angers B, Schlosser IJ (2007). The origin of *Phoxinus eos-neogaeus* unisexual hybrids. Mol Ecol.

[47] Bogart JP, Linton JE, Sandilands A (2017). A Population in limbo: unisexual salamanders (genus *Ambystoma*) decline without sperm-donating species. Herpetol Conserv Biol.

[48] Répartition géographique de la salamandre maculée Ambystoma maculatum. Atlas des Amphibien et Reptiles du Québec. 2009. http://www.atlasamphibiensreptiles.qc.ca/. Accessed 22 Jan 2014.

