# Supplementary material for: Influence of genome and bio-ecology on the prevalence of genome exchange in unisexuals of the Ambystoma complex
Source: BMC Evol Biol. 2018 May 31;18:82. doi: 10.1186/s12862-018-1200-7 (PMC5984407; doi:10.1186/s12862-018-1200-7)
Supplement: Supplementary file 1 — Table S1. Description of the environmental variables collected. Table S2. Primers and ranges of the loci used in the study. Sequence of the primers, specific range of alleles when known and reference are given. Table S3. Consensus score of the J-alleles for the five main genetic groups. Table S4. Allelic frequency for the 8 AFLP loci explaining the association to each group. Figure S1. Distribution of the sampling sites according to environmental conditions. (DOCX 241 kb) [file 12862_2018_1200_MOESM1_ESM.docx]

Table S1: Description of the environmental variables collected. Environmental variables were collected from May to August 2014, during the development period of blue-spotted salamander larvae. Some aquatic samples were taken with the YSI 556-MPS probe. The land samples were taken on two 20 x 20 m quadrats, located 50 m from the water body and 50 m apart.

| **Environmental variable** | **Units** | **Details** | |
| --- | --- | --- | --- |
|  |  |  |  |
| Conductivity | mS/cm | Probe YSI 556-MPS, average of the four months |  |
| Dioxygen concentration | mg/L | Probe YSI 556-MPS, average of the four months |  |
| Water pH | (pH) | Probe YSI 556-MPS, average of the four months |  |
| Oxydoreduction potential (ORP) | mV | Probe YSI 556-MPS, average of the four months |  |
| Type of pond substrate |  | Qualitative variable: 1 = humus ; 0 = clay |  |
| Water level | cm | Change of water level from a reference point (May = 0) |  |
| Presence/absence of water in summer |  | Qualitative variable: 1 = more than 15 cm at deepest point; 0.5 = less than 15 cm ; 0 = the pond is dry |  |
| Connexion with other water sources |  | Qualitative variable: 1 = the pond is linked to a stream ; 0 = the pond is isolated |  |
| Herbaceous presence |  | Qualitative variable: 1 = herbaceous plants are present in the pond ; 0 = absence of herbaceous plants |  |
| Tree presence |  | Qualitative variable: 1 = trees are present in the pond ; 0 = absence of trees |  |
| Canopy cover of the pond | % | Semiquantitative variable: 0%, 25%, 50%, 75%, 100% |  |
| Soil pH | (pH) | Average of the five data records for each quadrat for the four months |  |
| Type of forest substrate |  | Qualitative variable: 1 = humus ; 0 = clay |  |
| Drainage Rating |  | Semiquantitative variable: according to Emberger et al. 1968 |  |
| Percentage of conifers | % | Number of conifers/Number of trees in the quadrats |  |
| Canopy cover of the quadrats | % | Semiquantitative variable: 0% to 100%, classes of 10% |  |
| Time of the day | h | Number of hour since midnight |  |

Emberger L, Godron M, Daget P (1968). Code pour le relevé méthodique de la végétation et du milieu: Principes et transcription sur cartes perforées: Éditions du Centre national de la recherche scientifique.

Table S2: Primers, and ranges of the loci used in the study. Sequence of the primers, specific range of alleles when known and reference are given.

| **Locus** | **For-Primer** | **Rev-Primer** | **Total range** | **J-alleles range** | **L-alleles range** |  | **Reference** |
| --- | --- | --- | --- | --- | --- | --- | --- |
| AjeD13 | tttaaaccttaagagaaatcccag | ccatgttgtctgtctttgtgag | 200-228 | 200-228 | X |  | Julian *et al.* 2003b |
| AjeD23 | aaaacctctggagaaacatgag | gaacacaggctactaacaacagg | 197-253 | 197-229 | 229-253 |  | Julian *et al.* 2003b |
| AjeD84 | catgcatagcatcctgtgag | atatttaactgaggcctttggg | 148-196 | 148-196 | X |  | Julian *et al.* 2003b |
| AjeD84b | cccacacagtcaatccac | caagagcaaagatggttg | 150-234 | 150-198 | 182-234 |  | Present Study |
| AjeD94 | atatcccattccattgtttctg | atggacattcacatgatcacc | 142-242 | ? | ? |  | Julian *et al.* 2003b |
| AjeD283 | ttgcacccttggcagatg | tgtaatgggtcaggcaataatc | 118-194 | ? | ? |  | Julian *et al.* 2003b |
| AjeD294 | gttagtcgaactccggttgag | gtttctgtccgttgttgtctg | 234-250 | 234-250 | X |  | Julian *et al.* 2003b |
| AjeD346 | agcaggattagtgcttagatgc | tggcaatgtttacctaagagag | 180-200 | 180-200 | Unused* |  | Julian *et al.* 2003b |
| AjeD378 | ggcaaaccatattttccataac | agaaacctctgggtattaaggc | 220-276 | 220-276 | X |  | Julian *et al.* 2003b |
| AjeD422 | caaggtgctcaagttactgttc | caaattctgtacctgactgctg | 220-252 | 244-252 | 220-236 |  | Julian *et al.* 2003b |
| AmaD42 | gatggaaaatcaatcaagtgtg | taactagctgtcaatcgctctc | 133-257 | 133-193 | 165-257 |  | Julian *et al.* 2003a |
| AmaD42b | caatcaagtgtgacttcaagg | taactagctgtcaatcgctctc | 153-245 | X | 153-245 |  | Present study |

* L-alleles were not determined because PCR amplifications provided ambiguous genotyping results for this specific genome

Table S3: Consensus score of the J-alleles for the five main genetic groups. The more abundant allele is in bold character, whereas the other possible alleles are listed aside. Number of individuals carrying a given allele is indicated below the allele (total: 197 individuals). Boxes represent alleles close to the consensus genotype (± 2 mutation steps). The consensus genotype includes the most common allele for each loci.

|  |  | **AmaD42** | |  | **AjeD378** | | | |  | **AjeD13** | |  |  | **AjeD346** | | |  | **AjeD294** | |  | **AjeD422** | |  | **AjeD23** | |  | **AjeD84** | | |
| --- | --- | --- | --- | --- | --- | --- | --- | --- | --- | --- | --- | --- | --- | --- | --- | --- | --- | --- | --- | --- | --- | --- | --- | --- | --- | --- | --- | --- | --- |
|  |  |  |  |  |  |  |  |  |  |  |  |  |  |  |  |  |  |  |  |  |  |  |  |  |  |  |  |  |  |
| **A scores** |  | **137** | 133 |  | **232** | 220 |  |  |  | **212** | 216 |  |  | **180** | 184 |  |  | **246** | 242 |  | **244** |  |  | **225** |  |  | **194** | 198 | 190 |
| **n = 37** |  | **19** | 18 |  | **31** | 6 |  |  |  | **36** | 1 |  |  | **33** | 4 |  |  | **23** | 14 |  | **37** |  |  | **37** |  |  | **35** | 1 | 1 |
|  |  |  |  |  |  |  |  |  |  |  |  |  |  |  |  |  |  |  |  |  |  |  |  |  |  |  |  |  |  |
| **B scores** |  | **177** |  |  | **236** |  |  |  |  | **200** |  |  |  | **192** | 188 |  |  | **238** | 234 |  | **244** |  |  | **217** | 221 |  | **190** |  |  |
| **n = 19** |  | **19** |  |  | **19** |  |  |  |  | **19** |  |  |  | **11** | 8 |  |  | **18** | 1 |  | **19** |  |  | **11** | 8 |  | **19** |  |  |
|  |  |  |  |  |  |  |  |  |  |  |  |  |  |  |  |  |  |  |  |  |  |  |  |  |  |  |  |  |  |
| **C scores** |  | **177** |  |  | **244** |  |  |  |  | **224** | 228 |  |  | **184** | 188 | 180 |  | **246** |  |  | **244** |  |  | **197** |  |  | **190** | 194 |  |
| **n = 34** |  | **34** |  |  | **34** |  |  |  |  | **32** | 2 |  |  | **31** | 2 | 1 |  | **34** |  |  | **34** |  |  | **34** |  |  | **32** | 2 |  |
|  |  |  |  |  |  |  |  |  |  |  |  |  |  |  |  |  |  |  |  |  |  |  |  |  |  |  |  |  |  |
| **D (2n) scores** |  | **177** |  |  | **268** | 272 | 276 |  |  | **208** |  |  |  | **184** | 188 |  |  | **242** |  |  | **252** |  |  | **221** | 225 |  | **190** | 194 |  |
| **n = 27** |  | **27** |  |  | **15** | 11 | 1 |  |  | **27** |  |  |  | **21** | 6 |  |  | **27** |  |  | **27** |  |  | **16** | 11 |  | **22** | 5 |  |
|  |  |  |  |  |  |  |  |  |  |  |  |  |  |  |  |  |  |  |  |  |  |  |  |  |  |  |  |  |  |
| **D (3n) scores** |  | **177** | 181 |  | **264** | 260 | 268 | 272 |  | **208** | 204 | 212 |  | **188** | 184 | 192 |  | **250** | 246 |  | **248** | 252 |  | **229** | 225 |  | **194** | 190 |  |
| **n = 17** |  | **16** | 1 |  | **14** | 1 | 1 | 1 |  | **12** | 4 | 1 |  | **8** | 8 | 1 |  | **14** | 3 |  | **11** | 6 |  | **16** | 1 |  | **16** | 1 |  |
|  |  |  |  |  |  |  |  |  |  |  |  |  |  |  |  |  |  |  |  |  |  |  |  |  |  |  |  |  |  |
| **E scores** |  | **193** |  |  | **240** | 244 | 236 |  |  | **208** |  |  |  | **184** | 188 |  |  | **246** |  |  | **244** |  |  | **225** |  |  | **190** | 194 |  |
| **n = 63** |  | **63** |  |  | **57** | 3 | 3 |  |  | **63** |  |  |  | **61** | 2 |  |  | **63** |  |  | **63** |  |  | **63** |  |  | **61** | 2 |  |
|  |  |  |  |  |  |  |  |  |  |  |  |  |  |  |  |  |  |  |  |  |  |  |  |  |  |  |  |  |  |
| **Consensus score** | | **177** |  |  | **240** |  |  |  |  | **208** |  |  |  | **184** |  |  |  | **246** |  |  | **244** |  |  | **225** |  |  | **190** |  |  |
| **n_consensus_** |  | **96** |  |  | **57** |  |  |  |  | **102** |  |  |  | **125** |  |  |  | **123** |  |  | **153** |  |  | **112** |  |  | **136** |  |  |
| n_consensus_ ±8 bps | 97 | |  |  | 147 |  |  |  |  | 163 |  |  |  | 197 |  |  |  | 196 |  |  | 197 |  |  | 163 |  |  | 197 |  |  |
|  |  |  |  |  |  |  |  |  |  |  |  |  |  |  |  |  |  |  |  |  |  |  |  |  |  |  |  |  |  |

Table S4: Alleles frequency for the 8 AFLP loci explaining the association to each group.

| **group** | **gcta-429** | **gcta-262** | **gcag-317** | **gcag-309** | **gcag-263** | **gcaa-425** | **gcaa-370** |
| --- | --- | --- | --- | --- | --- | --- | --- |
| **A (n = 11)** | 0 | 0 | 0 | 0 | 1 | 0 | 0 |
| **B (n = 10)** | 0.8 | 0 | 0 | 0.8 | 0.7 | 1 | 0.1 |
| **C (n = 15)** | 0 | 0.87 | 0 | 0 | 0.87 | 1 | 1 |
| **D (n = 13)** | 1 | 1 | 1 | 0 | 0.92 | 0 | 0.92 |
| **E (n = 23)** | 0.65 | 0.13 | 0.04 | 0 | 0 | 0 | 0 |

Figure S1: Distribution of the sampling sites according to environmental conditions. Explanatory vectors have been enlarged by four times in order to show them clearly. Pie charts represent the proportion of each genetic groups (see Results: Origin of the J-haplome).
